# Heat-stable sublingual oxytocin tablets as a potential needle-free approach for preventing postpartum hemorrhage in low-resource settings

**DOI:** 10.1007/s13346-017-0471-7

**Published:** 2018-02-12

**Authors:** Changcheng Zhu, Marcus Estrada, Jessica White, Manjari Lal

**Affiliations:** PATH, 2201 Westlake Avenue, Suite 200, Seattle, Washington 98121 USA

**Keywords:** Sublingual administration, Freeze-drying, Heat-stable, Needle-free, Oxytocin

## Abstract

Postpartum hemorrhage is a major cause of mortality and morbidity related to childbirth in developing countries. The recommended treatment includes administration of oxytocin; however, oxytocin is a heat-labile protein, and it must be given as an intramuscular injection by skilled health care providers. To address these challenges, we developed a freeze-dried oxytocin fast-dissolving tablet (FDT) for sublingual (SL) needle-free administration. Using methods developed previously, we produced a robust FDT that maintained oxytocin stability at 40 °C, 75% relative humidity for 12 months. This formulation contains 9% sucrose, 1.5% (hydroxypropyl)methyl cellulose, 9% mannitol, 4% dextran, 1% carbomer, 1% sodium taurocholate, and 100 IU oxytocin. An in vitro study showed a > 30% reduction in tissue transepithelial electrical resistance after treatment with the oxytocin FDT, implying an increase in the permeability of the mucosal tissue to oxytocin. Anesthetized Yucatan miniature swine were administered a SL FDT, and blood was periodically collected for a pharmacokinetic study. Higher plasma concentrations were seen when larger SL doses were given. The maximum concentrations for SL and intramuscular doses in anesthetized pigs were 207 and 612 pg/mL, respectively. Whether the levels attained will be sufficient to elicit beneficial results in humans is yet to be determined. This study demonstrates the feasibility of our approach for developing a heat-stable oxytocin tablet that can be administered successfully via the SL route.

**Electronic supplementary material:**

The online version of this article (10.1007/s13346-017-0471-7) contains supplementary material, which is available to authorized users.

Postpartum hemorrhage (PPH)—loss of ≥500 mL of blood from the genital tract within 24 h of the birth of a baby—is a major cause of mortality, morbidity, and long-term disability related to pregnancy and childbirth, with most deaths occurring in developing countries [1]. Active management of the third stage of labor is the practice recommended by the World Health Organization (WHO) for reducing the incidence of PPH [2]; it consists of controlled cord traction, cord clamping, and administration of a uterotonic drug. WHO recommends oxytocin as the uterotonic drug of choice, to be given intramuscularly (IM) or intravenously for prevention or treatment of PPH. If oxytocin is not available or if skilled birth attendants are not present, WHO recommends oral misoprostol (600 mcg) [3]. This drug, however, has been associated with adverse side effects such as chills and fever (including hyperpyrexia), nausea and vomiting, diarrhea, and pain [4]. Oxytocin is a heat-labile protein and is typically administered as an IM injection by skilled health care providers—who are often not available at births in low-resource settings [5]. To address the challenges of heat instability and administration of oxytocin by injection, we conducted a feasibility study to develop a heat-stable, freeze-dried oxytocin fast-dissolving tablet (FDT) for sublingual (SL) needle-free administration. We then explored SL absorption of oxytocin from the FDT in a pharmacokinetic study in mini pigs.

Methods used to produce FDTs in our laboratory have been described previously [6–8]. Additional details on methods for the current study are in Online Resource 1. Briefly, aqueous excipients—including stabilizers, mucoadhesive polymers, and permeation enhancers—were combined in various ratios and mixed with oxytocin. These liquid formulations were lyophilized in preformed blister sheets to form FDTs directly in the cavities. All formulations contained 9% sucrose and 9% mannitol. Other components tested were the following: (hydroxypropyl)methyl cellulose (HPMC), polyvinylpyrrolidone (PVP-40), dextran, carbomer, sodium glycodeoxycholate, chitosan, L-α-phosphatidylcholine, polyarginine, sodium taurocholate, and decyl trimethyl ammonium bromide. To assess the content of oxytocin in the tablets after various physicochemical stability evaluations, reverse-phase high-performance liquid chromatography (HPLC) was performed [6].

We designed 13 formulations as FDTs and performed physicochemical screening to down-select candidates for further testing; compositions of all 13 are provided in Online Resource 2. Tablets that melted, retracted from the edge of the blisters, left a residue upon removal, or presented a non-homogenous appearance were discarded. Five formulations were discarded after the first screening of physical attributes, and further testing was performed on the remaining formulations.

Rapid disintegration of the FDT is essential for maximizing absorption of available oxytocin into systemic circulation, to stop postpartum bleeding as quickly as possible; it also reduces involuntary dislodging and swallowing of the tablet. Since there is limited volume of saliva in the oral cavity, FDT disintegration was tested in conditions simulating resting oral fluid volumes [9]. We placed 1.25 mL of pooled human saliva in a petri dish, added a tablet, and observed disintegration. Five of the remaining eight FDT formulations disintegrated within 10 s, losing physical integrity and leaving no particulate matter visible to the naked eye. These candidates were then assayed by HPLC for oxytocin content, to measure loss in drug content encountered during the freeze-drying process. The formulation with the least process loss that also maintained tablet properties was identified as the lead candidate and taken forward for further testing. This formulation—containing 9% sucrose, 1.5% HPMC, 9% mannitol, 4% dextran, 1% carbomer, and 1% sodium taurocholate—formed a robust white tablet weighing 110 to 130 mg and incorporating approximately 100 IU oxytocin (Fig. 1).

**Fig. 1:**
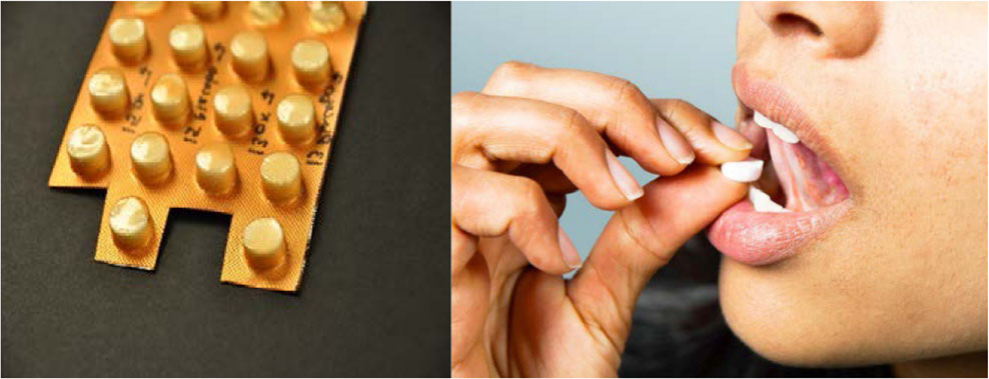
Heat-stable sublingual oxytocin fast-dissolving tablets in **a** blister packs and **b** simulated use. Freeze-drying took place directly in preformed blister sheets

Since oxytocin is reportedly sensitive to heat and thus requires a cold chain, one of our objectives was to develop a heat-stable form of oxytocin that could be transported and stored in low-resource areas without requiring expensive cold chain equipment. To test for oxytocin stability during long-term storage under simulated field conditions, sealed blister sheets of oxytocin FDTs in secondary foil packaging were stored in 40 °C, 75% relative humidity chambers for 12 months. Once a month, tablets were tested for oxytocin content by HPLC and for any changes in appearance such as shrinkage or collapse. The oxytocin content remained stable within acceptable limits of 90 to 110% potency for the duration of the entire study (Fig. 2). The liquid oxytocin control showed greater than 30% loss in oxytocin content at the end of 1 month under same conditions (data not shown). The residual moisture in the tablet was less than 2.5% at the end of the 12-month storage study (data not shown), and no visual change was observed in physical integrity of the FDTs.

**Fig. 2:**
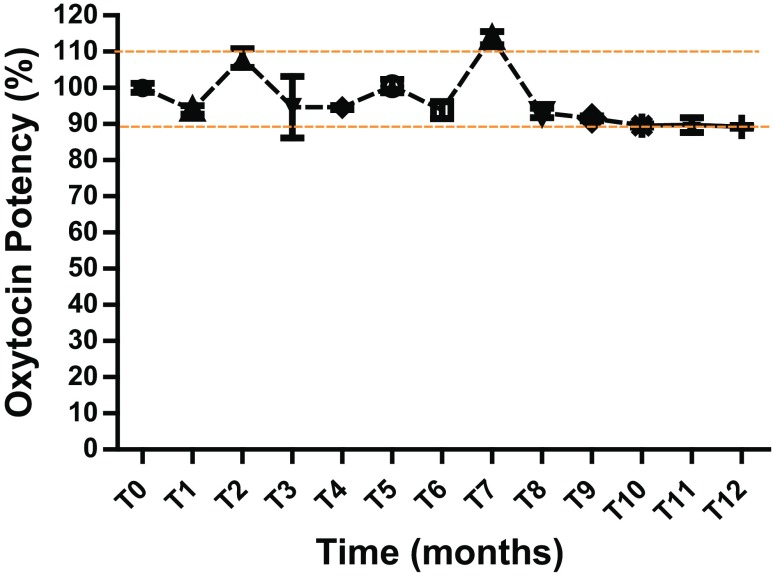
Oxytocin content over a 12-month storage period as percentage of the amount present at time zero. Oxytocin tablets in blister sheets with an aluminum foil lid were sealed in aluminum foil sachets and placed in a 40 °C, 75% relative humidity chamber. Content was measured by high-performance liquid chromatography at monthly time points

After oxytocin is released, post disintegration of the FDT, it is important that it be rapidly absorbed through the underlying mucosal epithelium into systemic circulation. This was achieved by incorporating permeation enhancers into our formulations; sodium taurocholate, a bile salt, was the enhancer in our lead candidate formulation. Transepithelial electrical resistance (TEER) measurements were performed on human buccal tissue samples after exposure to FDTs to determine changes in permeability of the oral mucosa upon contact with this formulation. Method details are in Online Resource 1. Briefly, tissue resistance measurement chambers and tissue samples were equilibrated and washed; then, resting TEER measurements were taken when phosphate-buffered saline was placed on the apical tissue surface. Tissues were washed again and treated with either saliva or saliva containing a disintegrated oxytocin FDT. After incubation, tissues were washed and TEER measurements again recorded. The difference in resistance between tissue treated with saliva and tissue treated with saliva containing the FDT oxytocin formulation was calculated. A reduction in the TEER of > 30% (from 308.8 ± 9.4 to 154.8 ± 10.6 Ω/cm^2^) was seen in the tissue after treatment with the oxytocin FDT, implying that this formulation may increase the permeability of the mucosal tissue to oxytocin.

Since the oxytocin FDTs are intended to be administered under the tongue, we confirmed the stability of oxytocin in saliva prior to initiation of the pharmacokinetic study. The FDTs were incubated in pooled human saliva maintained at 37 °C for an hour along with unformulated oxytocin as a control. The results from HPLC analysis indicated none to minimal proteolytic degradation of oxytocin in saliva.

Next, a crossover pharmacokinetic study with 24-h washout was conducted to determine oxytocin plasma levels after SL administration and to compare with the standard IM route in female mini pigs, chosen due to the similarity in oral cavity architecture with humans. Ten anesthetized female, non-pregnant Yucatan miniature swine, *Sus scrofa* 3 to 5 months old and weighing 15 to 20 kg were administered an oxytocin FDT under the tongue. For the control, mini pigs received 20 IU of oxytocin in a 1 mL IM injection into the right hind limb. To account for drug loss due to involuntary swallowing and dilution by excess saliva, a 20 to 30× higher dose was tested via the SL route. Although this is a high dose, levels greater than 1000 IU reportedly have been tested in women via the buccal route for induction of labor, without any adverse events [10]. Blood was collected from both groups at several time points up to 60 min after administration, via previously implanted vascular access ports, and plasma was obtained for analysis. A commercial enzyme-linked immunosorbent assay kit was used to detect oxytocin in the plasma. See Online Resource 1 for further details.

Evaluation of SL uptake of oxytocin showed that a larger dose appeared to be associated with higher plasma levels (Fig. 3), although there was significant variability among animals within each dosing group. We attribute the variability to limitations of the sublingual route of dosing: individual animals may secrete different amounts of saliva; the placement of the tablet may differ; and it is difficult to ensure that the liquid containing the disintegrated tablet remains under the tongue. Thus, the amount of drug loss into the oral cavity varied among animals. Plasma oxytocin levels were lower with SL administration than via the IM route in anesthetized pigs (Fig. 3), likely because of loss to the oral cavity with subsequent swallowing. The maximum plasma concentration for SL dosing was 50.5 and 207 pg/mL for dosages of 400 and 600 IU, respectively. For IM administration to anesthetized pigs, the maximum concentration was 612 pg/mL.

**Fig. 3:**
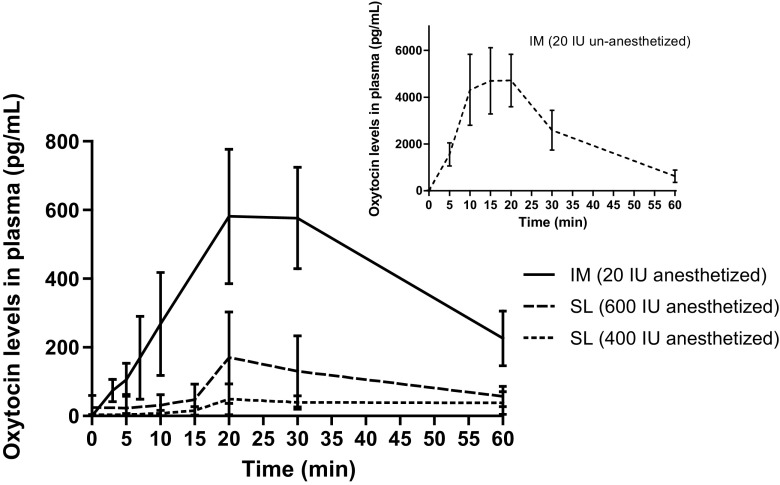
Oxytocin concentration in pig plasma after intramuscular injection or sublingual administration of fast-dissolving tablets. Concentration was measured by high-performance liquid chromatography. Abbreviations: *IM* intramuscular, *pg/mL* picograms per milliliter, *SL* sublingual

For IM administration, the plasma levels of oxytocin obtained in unanesthetized pigs were significantly higher than anesthetized pigs (separate study). One explanation could be the effect of anesthetic agents, which can depress blood pressure. Local blood flow is a strong determinant of the rate of absorption, since it continuously maintains the concentration gradient necessary for passive diffusion to occur. It is possible that lower plasma levels seen for SL administration as well as for IM administration in anesthetized pigs can be attributed to reduced blood flow in sedated animals.

Administration of drugs to the oral cavity is generally acceptable to patients, and medications can be self-administered or given by a caregiver with minimal training since no needle injection is necessary. Freeze-drying (lyophilization) is a well-established process that is traditionally used for stabilizing labile substances or processing pharmaceuticals of biological origin, such as proteins, serum, vaccines, peptides, drugs, or liposomes, which are often administered parenterally [11]. A consideration for oxytocin, a small protein, is protection from denaturation due to ice formation during the freeze-drying process. The main function of stabilizing excipients such as sucrose in our formulation is to act as cryoprotectants and to maintain stability during storage and in solution after reconstitution. Excipients also are added to optimize the final product appearance, which should have no phase separation (indicative of stability), should be rapidly dissolvable, and have good physical handling properties. For this purpose, bulking agents such as mannitol and dextran were added, with mannitol assisting in good structural stability and handling properties.

Because of the rich blood supply and high permeability of the SL mucosa, administration via this route can result in rapid onset of action [10]. Despite the fact that at the time of administration, the available volume of saliva under the tongue is small, the area is continuously washed by saliva generated in response to external stimuli; therefore, for rapid and significant drug absorption, oxytocin must be available quickly at the site and must have a prolonged retention at the mucosal surface. The human saliva in our testing hydrated the polymers in the tablet, causing disintegration of the FDT in the absence of any mechanical force, resulting in a soft, viscous, gel-like mass, characteristic of a mucoadhesive polymer. HPMC and carbomer, both mucoadhesive polymers, are the components of the FDT that provided these characteristics, which are necessary for increased local retention, maximizing the time for absorption.

The feasibility study reported here demonstrates successful development of heat-stable oxytocin in a freeze-dried tablet for SL administration. While our pharmacokinetic study in pigs provides evidence for SL absorption of oxytocin into plasma, whether the oxytocin levels attained via SL administration will be sufficient to elicit beneficial results in humans is yet to be determined. The clinical potential of this presentation to prevent PPH will need to be addressed by first conducting an exploratory pharmacokinetic study in healthy non-pregnant women. Our study opens the possibility for needle-free administration of oxytocin, which would be especially useful in low-resource areas with a scarcity of skilled health workers.

## Electronic supplementary material

ESM 1 (PDF 114 kb)

ESM 2 (PDF 100 kb)
