# Supplementary material for: Heat-stable sublingual oxytocin tablets as a potential needle-free approach for preventing postpartum hemorrhage in low-resource settings
Source: Drug Deliv Transl Res. 2018 Feb 12;8(3):853–6. doi: 10.1007/s13346-017-0471-7 (PMC5937893; doi:10.1007/s13346-017-0471-7)
Supplement: Supplementary file 1 — (PDF 114 kb) [file 13346_2017_471_MOESM1_ESM.pdf]

Supplementary Materials

Manuscript: Heat-stable sublingual oxytocin tablets as a potential needle-free approach for preventing postpartum hemorrhage in low-resource settings

Online Resource 1
Materials and methods

Corresponding Author: Manjari Lal, PhD

Author address:
PATH, 2201 Westlake Avenue, Suite 200, Seattle, Washington 98121, USA
E-mail address: mlal@path.org

Formulation components

The following materials were used in formulation development: carbomer, sodium taurocholate, sodium glycodeoxycholate, decyl trimethyl ammonium bromide, L-α-phosphatidylcholine, and polyarginine (all from Sigma Aldrich, St. Louis, MO, USA), and carbomer (Noveon AA-1 polycarbophil; Lubrizol Advanced Materials, Inc, Cleveland, OH, USA). Other components were chitosan (Acros Organics/Thermo Fisher Scientific, NJ, USA); sucrose and mannitol (J.T. Baker/Fisher Scientific, Loughborough, England); and polyvinylpyrrolidone (PVP-40), (hydroxypropyl)methyl cellulose (HPMC), and dextran (TCI America, Portland, OR, USA). Oxytocin was from Hemmo Pharmaceuticals Inc., Mumbai, India.

Preparation of formulations

Formulations were prepared by mixing one part of 4X oxytocin solution with three parts of 4/3X placebo solution. To make 50 mL of 4/3X placebo solution, 4/3of the final required amount of permeation enhancer and of carbomer were mixed with 15 mL of water and stirred at 400 rpm for 30 minutes in a 100 mL Erlenmeyer flask. In a separate 50 mL Erlenmeyer flask, 4/3 of the final required amount of HPMC was dissolved in 10 mL of boiling water and then 10 mL of cold water was added, with stirring at 400 rpm. The HPMC solution was poured into carbomer mixture and washed twice with 2 mL of water to recover any remaining contents. The other excipients were weighed and added directly to the Erlenmeyer flask containing carbomer and HPMC. The final placebo mixture was stirred overnight and brought to 50 mL using a 50 mL volumetric flask.

Four times the final dose of oxytocin was dissolved in water to make a 4X concentration of oxytocin solution. One part of 4X oxytocin solution was mixed with three parts of 4/3X placebo solution and stirred at 300 rpm for 5 minutes to make the final formulations.

High-performance liquid chromatography

Reverse-phase high-performance liquid chromatography (HPLC) was performed in the Dionex™ UltiMate™ UHPLC 3000 system at 30°C (Thermo Fisher Scientific, Sunnyvale, CA, USA). An Acclaim® RSLC 2.2 µm column (Thermo Fisher; 2.2 µm C18, 120Å, 2.1 x 100 mm) protected by Acclaim® 120, C18, 5 µm guard cartridges (2.1 x 10 mm) was used with a mobile phase of 65% 0.1 M monosodium phosphate/35% acetonitrile (Sigma) in water (1:1), pH 3.5, at a flow rate of 0.4 mL/minute. The detection wavelength was 210 nm, and 10 µL of sample was injected. Data were analyzed using the Dionex™ Chromeleon™ Chromatography Data System.

Forming of blister sheets

Blister sheet forming and sealing were performed on a B2FS thermoforming and sealing machine (Applied Engineering Corporation, Garfield, NJ, USA). Polyvinyl chloride blister sheets (Delta Circle Industries, Richmond, VA, USA) were cut into 3.4 x 4 inch pieces. The pieces were thermoformed to obtain 1 mL cavities with a diameter of 15 mm. Each blister sheet contained 12 cavities. Process parameters for forming were as follows: preheat time 3.5 seconds, forming time 3.5 seconds, sealing time 3.5 seconds, temperature 150±5°C, and air pressure 80 psi.

Lyophilization and sealing

Formulations containing oxytocin were loaded into preformed blister sheets on lyophilization trays using a HandyStep® electronic repeating pipette (BrandTech Scientific, Essex, CT, USA). Lyophilization was performed on a Millrock Laboratory Freeze Dryer (Millrock Technology, Kingston, NY, USA) using a highly conservative cycle which lasted for 4 days. The condenser temperature was set at –70°C, and the vacuum was set at 100 mTorr and kept constant throughout the process. The entire cycle lasted 94 hours.

Fast-dissolving tablets (FDTs) in the blister sheets were removed from the freeze dryer and immediately sealed with a peelable foil lid (Delta Circle Industries, Richmond, VA, USA) using a B2FS thermoforming and sealing machine at 155°C. The sealing time was 3.5 seconds at a temperature of 150±5°C and air pressure of 80 psi. Secondary packaging was performed by placing the sealed blister sheets in aluminum foil sachets (Pharmaceutical Packaging Services, Richmond, VA, USA), followed by sealing (Medivac sealer, ALINESYS.com, Los Angeles, CA, USA).

Moisture content analysis

The moisture content of the FDTs was determined by methanol extraction and coulometric Karl Fischer titration in a dry environment. Anhydrous methanol was added to each FDT, and the vessel was agitated for 1 minute. The dry methanol was in contact with the FDT for 20 minutes and then was analyzed for moisture content using a C20 Karl Fischer Coulometric Titrator (Mettler-Toledo LCC, Columbus, OH, USA).

Permeability measurements by transepithelial electrical resistance

Transepithelial electrical resistance (TEER) measurements were performed on human buccal tissue samples (MatTek, Ashland, MA, USA) before and after exposure to oxytocin FDTs in a cell culture hood using aseptic technique. We used an AssayReady™ Caco-2 6-well kit (MB Biosciences LLC, Chestnut Hill, MA, USA) with Dulbecco’s Modified Eagle Medium, low glucose, pyruvate, penicillin-streptomycin (10,000 U/mL) (Gibco/Thermo Fisher Scientific), and 10X Hank’s balanced salt solution (JT Baker/Thermo Fisher, Bothell, WA, USA).

Measurements were taken following the protocol provided by MatTek. In brief, the following procedures were performed. An epithelial volt/ohm meter for TEER (EVOM2) and EndOhm tissue resistance measurement chambers (World Precision Instruments, Sarasota, FL, USA) were used for TEER measurements after equilibration in phosphate-buffered saline (PBS) for 20 minutes at room temperature. Tissues were equilibrated in 3 mL of prewarmed medium in a 6-well cell culture plate at 37°C for 1 hour. After equilibration, tissues were washed twice with 1.5 mL of prewarmed PBS followed by one wash with 1.5 mL of sterile water. Resting TEER measurements were taken by placing tissues in a 6-well plate containing prewarmed PBS, adding 3 mL of PBS to the apical tissue surface, and measuring resistance with the EVOM2. Tissues were washed as described above prior to tablet treatment. Tissues were treated on the apical surface with either 1 mL of pooled human saliva (Lee Biosolutions, Maryland Heights, MO, USA) or 1 mL of saliva with a disintegrated FDT, and incubated at 37°C for 20 minutes. After incubation, tissues were washed as described above and TEER measurements were recorded in prewarmed PBS using the same method as for resting measurements. The difference in the TEER was determined by subtracting

the resting TEER measurement from the final TEER measurement. A reduction in the TEER indicates increased permeability of oral tissue after treatment. All treatments were performed in duplicate on separate tissue samples.

Disintegration testing: Non-sink method

Because only a small volume of saliva is available in the oral cavity, we performed non-sink disintegration testing by adding 1.25 mL of pooled human saliva to each testing well in a 12-well cell culture plate at room temperature. An oxytocin tablet was dropped into the center of the test well and observed at different time points (0, 10, 20, 30, 50, 60, 90 seconds) for complete disintegration. The average disintegration time for n=3 FDTs was reported.

Saliva stability study

Oxytocin FDTs were reconstituted into 1 mL of pooled human saliva, and 50 uL of the obtained formulation was aliquoted into four different vials. Two aliquots were diluted with the HPLC mobile phase and centrifuged immediately. The supernatants were loaded for HPLC analysis for the initial (time zero) time point. The other two vials were set into a heating block preheated to 37°C. After 1 hour, aliquots from these vials were diluted with the HPLC mobile phase liquid and centrifuged immediately. The supernatants were loaded for HPLC analysis at the 1-hour time point. The oxytocin recovery rate for each formulation was calculated as the oxytocin content at time 1-hour divided by the content at time zero.

12-month stability study

Sealed blister sheets containing oxytocin FDTs were stored in a 40°C, 75% relative humidity chamber for 12 months. At each monthly time point, blister sheets were removed and tablets tested for recovery of oxytocin by HPLC.

Pharmacokinetic study

The pharmacokinetic data were derived from ten non-pregnant female Yucatan miniature swine, Sus scrofa, aged 3 to 5 months, weighing 15 to 20 kg (Sinclair Bio-Resources, LLC, Auxvasse, MO, USA). Ten experimental pigs received a sublingual (SL) tablet with 400 IU of oxytocin, and ten received SL tablets with 600 IU oxytocin. Ten control pigs received 20 IU of oxytocin in a 1 mL intramuscular (IM) injection.

Test material	Route of administration	Dose level (IU/animal)	Dose administered
Formulation 4	Sublingual	600	1 tablet
Formulation 4	Sublingual	400	1 tablet
Control	Intramuscular	20	1 mL of 20 IU/mL

Animals were anesthetized with dexmedetomidine (0.04 mg/kg) mixed with midazolam (0.2 mg/kg), and anesthesia was reversed with atipamezole (up to 0.2 mg/kg, IM injection) and flumazenil (0.02 mg/kg, IM injection). The animals were supplemented with isoflurane administered via face mask, if necessary. The FDTs were administered once on days 1, 3, 8, 15, and 18. Following placement of each tablet, 0.5 mL of purified water was administered to aid in dissolution of the tablet. The control was administered to the appropriate animals via IM injection into the right hind limb (biceps femoris) once on day 22. There was a minimum 1-day washout period after each dose.

Blood samples were collected at 0, 5, 10, 15, 20, 30, and 60 minutes after dose administration. About 3 mL of whole blood was collected via vascular access ports implanted prior to the initial dose administration. Blood samples were collected into sterile, prechilled 3 mL vacutainer tubes containing K2EDTA as the anticoagulant, preloaded with

30 µL of aprotinin (10 mg/mL concentration). The blood samples were placed into the tubes and inverted multiple times to ensure proper mixture prior to placing on ice packs until processing. All blood samples were centrifuged at ~3,000 rpm for ~15 minutes at ~ 4°C to separate the plasma. Plasma samples were split into primary and back-up samples (a minimum of 500 µL per sample) and immediately placed on dry ice. The plasma samples were stored frozen at approximately –70°C until analysis.

ELISA for plasma sample analysis

A commercial enzyme-linked immunosorbent assay (ELISA, Phoenix Pharmaceuticals, Inc., Burlingame, CA, USA) was used to detect oxytocin in pig plasma. A standard curve was generated by a 2.5-fold sequential dilution of starting oxytocin concentration of 1,000 pg/mL in assay buffer, for a total of eight points on the curve. Three concentrations of oxytocin (240 pg/mL, 60 pg/mL, 15 pg/mL) were spiked into pig plasma as high-, medium-, and low-quality control samples. Pharmacokinetic and quality control samples were extracted using an Arbor Assays extraction solution according to the protocol provided by the manufacturer (Arbor Assays, Ann Arbor, MI, USA). The extracted samples were centrifuged for 20 minutes at 21,000x g at 4°C. The supernatants were separated and dried using a SpeedVac at 30°C for 3.5 hours. Dried samples were reconstituted in 0.3 mL of assay buffer and proceeded for analysis, with or without dilution, per the testing requirements for the collected plasma samples.
