# Supplementary material for: Heat-stable sublingual oxytocin tablets as a potential needle-free approach for preventing postpartum hemorrhage in low-resource settings
Source: Drug Deliv Transl Res. 2018 Feb 12;8(3):853–6. doi: 10.1007/s13346-017-0471-7 (PMC5937893; doi:10.1007/s13346-017-0471-7)
Supplement: Supplementary file 2 — (PDF 100 kb) [file 13346_2017_471_MOESM2_ESM.pdf]

Supplementary Materials

Manuscript: Heat-stable sublingual oxytocin tablets as a potential needle-free approach for preventing postpartum hemorrhage in low-resource settings

Online Resource 2
Compositions of fast-dissolving tablets tested for physiochemical properties

Corresponding Author: Manjari Lal, PhD

Author address:
PATH, 2201 Westlake Avenue, Suite 200, Seattle, Washington 98121, USA
E-mail address: mlal@path.org

Formulation	Compositions (wt%)*	Physicochemical properties
1	9% sucrose, 1.5% HPMC, 9% mannitol, 4% dextran, 0.2% carbomer, 1% sodium glycodeoxycholate	Low-quality tablet
2	9% sucrose, 1.5% HPMC, 9% mannitol, 4% dextran, 0.2% carbomer, 0.2% chitosan	Robust tablet, instant disintegration (<10 seconds), some insoluble matter
3	9% sucrose, 1.5% HPMC, 9% mannitol, 4% dextran, 0.2% carbomer,1% L-α-phosphatidylcholine	Robust tablet, instant disintegration (<10 seconds)
**4[†]**	**9% sucrose, 1.5% HPMC, 9% mannitol, 4% dextran, 1% carbomer, 1% sodium taurocholate**	**Robust tablet, instant disintegration (<10 seconds)**
5	9% sucrose, 4% PVP-40, 9% mannitol, 2% dextran, 1% sodium glycodeoxycholate	Low-quality tablet
6	9% sucrose, 4% PVP-40, 9% mannitol, 2% dextran, 0.05% chitosan	Low-quality tablet
7	9% sucrose, 4% PVP-40, 9% mannitol, 2% dextran, 0.1% chitosan	Robust tablet, instant disintegration (<10 seconds)
8	9% sucrose, 4% PVP-40, 9% mannitol, 2% dextran, 1% DTAB, 0.05% chitosan	Robust tablet, instant disintegration (<10 seconds)
9	9% sucrose, 4% PVP-40, 9% mannitol, 2% dextran, 1% DTAB	Robust tablet, instant disintegration (<10 seconds)
10	9% sucrose, 4% PVP-40, 9% mannitol, 2% dextran, 1% L-α-phosphatidylcholine	Low-quality tablet
11	9% sucrose, 4% PVP-40, 9% mannitol, 2% dextran, 0.5% polyarginine	Low-quality tablet
12	9% sucrose, 1.5% HPMC, 9% mannitol, 2% dextran, 0.1% chitosan	Robust tablet, instant disintegration (<10 seconds), some insoluble matter
13	9% sucrose, 1.5% HPMC, 9% mannitol, 2% dextran, 0.05% chitosan	Robust tablet, instant disintegration (<10 seconds), some insoluble matter

Abbreviations: DTAB, decyl trimethyl ammonium bromide; HPMC, (hydroxypropyl)methyl cellulose; PVP-40, polyvinylpyrrolidone.

[*] All FDTs also contained 100 IU oxytocin.

[†]Formulation 4 was the lead candidate from this study, based on physiochemical properties and further testing described in the text.
